# Study on performance of magnetic fluorescent nanoparticles as gene carrier and location in pig kidney cells

**DOI:** 10.1186/1556-276X-8-127

**Published:** 2013-03-15

**Authors:** Yan Wang, Haixin Cui, Changjiao Sun, Wei Du, Jinhui Cui, Xiang Zhao

**Affiliations:** 1 Institute of Environment and Sustainable Development in Agriculture, Chinese Academy of Agricultural Sciences, Beijing, 100081, China

**Keywords:** magnetic, fluorescent nanoparticles, gene carrier, location, 75.50.-y, 81.07.-b, 87.85.Rs

## Abstract

We evaluated the performance of green fluorescent magnetic Fe_3_O_4_ nanoparticles (NPs) as gene carrier and location in pig kidney cells. When the mass ratio of NPs to green fluorescent protein plasmid DNA reached 1:16 or above, DNA molecules can be combined completely with NPs, which indicates that the NPs have good ability to bind negative DNA. Atomic force microscopy (AFM) experiments were carried out to investigate the binding mechanism between NPs and DNA. AFM images show that individual DNA strands come off of larger pieces of netlike agglomerations and several spherical nanoparticles are attached to each individual DNA strand and interact with each other. The pig kidney cells were labelled with membrane-specific red fluorescent dye 1,1^′^-dioctadecyl-3,3,3^′^,3^′^-tetramethylindocarbocyanine perchlorate and nucleus-specific blue fluorescent dye 4^′^,6-diamidino-2-phenylindole dihydrochloride. We found that green fluorescent nanoparticles can past the cell membrane and spread throughout the interior of the cell. The NPs seem to locate more frequently in the cytoplasm than in the nucleus.

## Background

Viral vectors have been extensively investigated as the most efficient and commonly used delivery modalities for gene transfer [1,2]. However, issues of immune response to viral proteins remain to be addressed. Recent efforts have focused on developing non-viral gene transfer systems, and significant progress has been made in this area [3-5]. Non-viral delivery systems have potential advantages such as ease of synthesis, cell targeting, low immune response, and unrestricted plasmid size. Among non-viral delivery systems, nanoparticle-based systems have excited great interest among scientists due to the active surface properties, strong penetrability with small size, protective effect on genes, and low toxicity [6-10].

However, a limitation of the non-viral delivery technologies is the lack of an intrinsic signal for long-term and real-time imaging of gene transport and release. Such imaging could provide important information on rational design of gene carriers. Currently, organic fluorophores are used to label gene delivery [11], but the photobleaching problem prevents long-term tracking.

With the rapid development of surface chemical modification method and nanobiotechnology, nanoparticle-based non-viral-mediated systems will help to achieve the ability to traceable, safe, efficient, and targeted DNA delivery. Qi and Gao reported that a new quantum dot-amphipol nanocomplex allows efficient delivery and real-time imaging of siRNA in live cells [12], but the nanocomplex cannot drive genes with magnetic targeting. Electron-dense gold nanoparticles (NPs) are reported to provide the highest imaging resolution in fixed cells due to their visibility under a transmission electron microscope [13], but they do not allow real-time imaging of live cells.

Here, we report green fluorescent magnetic Fe_3_O_4_ nanoparticles as gene carrier and evaluated their performance and location in pig kidney cells. This work focused primarily on evaluating performance of the green fluorescent magnetic Fe_3_O_4_ nanoparticles as gene carrier in mammalian somatic cells, which is significant research for their further application in animal genetics and breeding. Magnetic nanoparticle gene carriers, as non-viral carriers, are not easily digested; have superparamagnetism, higher DNA carrying capacity, and powerful penetration ability; are convenient and low cost; and can drive target genes to express highly under external magnetic field. Moreover, magnetic fluorescent nanoparticles could provide an intrinsic signal for imaging of gene transport in the cells, which makes them a potential application prospect in animal genetics and breeding.

## Methods

### Experimental materials

In this study, the green fluorescent magnetic Fe_3_O_4_ nanoparticles were purchased from Chemicell (25 mg/mL, Berlin, Germany), which is enveloped in the matrix of poly-(dimethylamin-*co*-epichlorhydrin-*co*-ethylendiamin). The amine group is the functional group for conjugation with biomolecules.

We used a plasmid containing a green fluorescent protein gene as model plasmid to investigate the binding ability of nanoparticles with plasmid DNA. The green fluorescent protein plasmid, which expresses enhanced green fluorescent protein under the control of the cytomegalovirus promoter, was purchased from BD Biosciences Clontech (Palo Alto, CA, USA). The plasmid DNA was amplified in *Escherichia coli* bacteria and then isolated and purified using the Vigorous Plasmid Maxprep Kit (Beijing, China) according to the manufacturer’s instruction. Porcine Kidney-15 (PK-15) cells were provided by the Institute of Animal Sciences, Chinese Academy of Agricultural Sciences.

### Agarose gel electrophoresis of NP-DNA complexes

To test whether magnetic nanoparticles can bind DNA plasmid effectively, the complexes formed by nanoparticles and plasmid DNA were examined by agarose gel electrophoresis (Gel Doc™ EZ, Bio-Rad Laboratories, Inc., Hercules, CA, USA) with various mass ratios of nanoparticles to plasmid DNA (1:1, 1:8, 1:16, 1:24, 1:40, 1:64). After 30 min of incubation at room temperature for the complex formation, the samples were electrophoresed on a 1% (*w*/*v*) agarose gel and stained in an ethidium bromide solution (0.5 μg/mL). The location of the DNA was analyzed on a UV illuminator.

### Investigation of binding mechanism by atomic force microscopy

Atomic force microscopy (AFM; Multimode NS-3a, Veeco, Santa Barbara, CA, USA) was employed to study the morphology and microstructure of DNA, NPs, and NP-DNA complex. The images were used to analyze the binding mechanism between plasmid DNA and NPs. To prepare the NP-DNA complex, the plasmid DNA and NPs were mixed and incubated for 30 min. The final samples were dropped on fresh sheets of glass and air-dried. The combination mechanism of NPs and DNA can be investigated by the AFM images.

### The location of NPs in the cells

In order to observe visually the location of NPs in the cells, the pig kidney cells (PK-15 cells) were labelled with membrane-specific red fluorescent dye 1,1^′^-dioctadecyl-3,3,3^′^,3^′^-tetramethylindocarbocyanine perchlorate (DiI) and nucleus-specific blue fluorescent dye 4^′^,6-diamidino-2-phenylindole dihydrochloride (DAPI). In detail, PK-15 cells were plated in glass-bottom Petri dishes, loaded with membrane-specific fluorescent dye DiI for 10 min first and then the blue fluorescent dye DAPI for 5 min. Next, the original solution of green fluorescent magnetic Fe_3_O_4_ nanoparticles was diluted. A 0.5-μL diluted solution of magnetic nanoparticles was added into the Petri dishes with a concentration of 0.002 μg/μL, and then the labelled cells were incubated with green fluorescent magnetic Fe_3_O_4_ nanoparticles under the drive of an external magnetic field for 30 min. The location of NPs in the cells was measured by confocal laser scanning microscopy (A1R-Si, Nikon, Yokohama, Japan).

## Results and discussion

### Agarose gel electrophoresis of NP-DNA complexes

**Figure 1:**
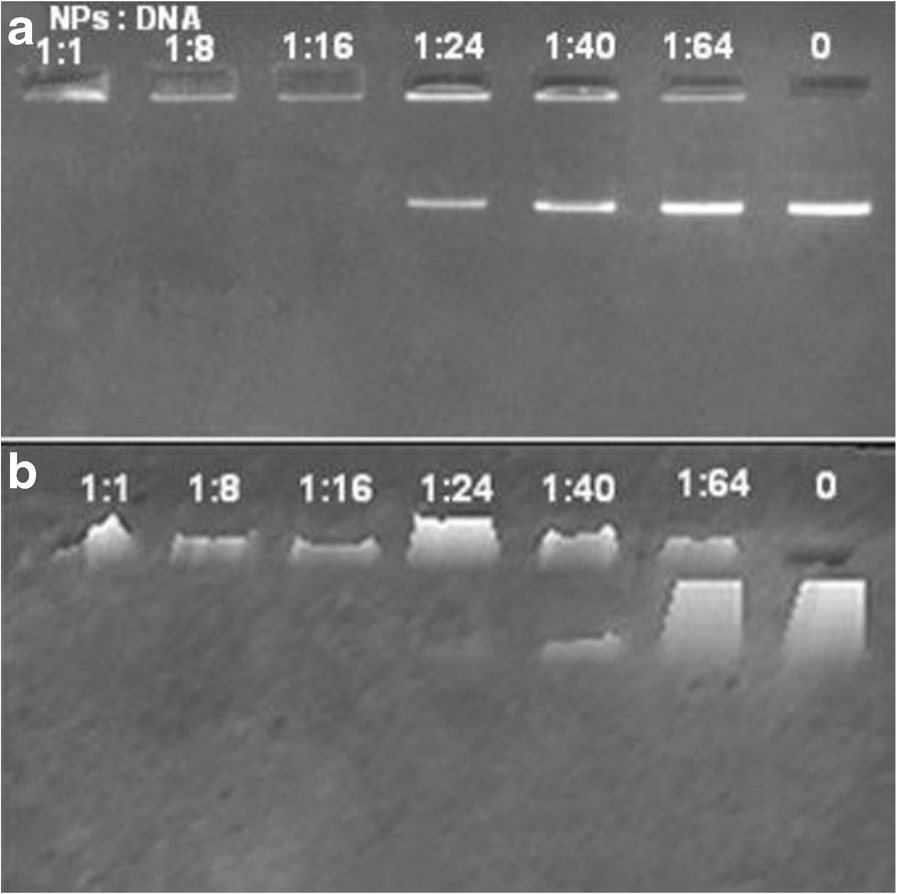
**Agarose gel electrophoresis of plasmid NP-DNA complex and corresponding three-dimensional projection plot of band intensities. **(**a**) Agarose gel electrophoresis of plasmid DNA and NP complex with various DNA/NP mass ratios. (**b**) Corresponding three-dimensional projection plot of band intensities of the same gel as in (**a**). Results were obtained using image analysis software. Plasmid DNA and various amounts of NPs were mixed, and the mass ratio is indicated above each lane (pure plasmid DNA in the rightmost lane).

Formation of complexes of plasmid DNA with NPs was evaluated by agarose gel electrophoresis with various ratios of NPs to plasmid DNA. Figure 1a shows the gel electrophoresis image results for the NP-DNA complexes, which were formed by electrostatic interactions. Figure 1b shows a three-dimensional projection plot of the intensities of the same gel as in Figure 1a. As shown in Figure 1a, migration of the DNA on the gel gradually decreases when the concentration of NPs increases due to charge neutralization and increased molecular size of the complexes. The intensity of various bands can be viewed by transforming the corresponding gel image to a solid three-dimensional model. From the three-dimensional projection in Figure 1b, we can evaluate and observe visually the variation tendency of the intensity for various bands. The analysis of an electrophoresis gel can be both qualitative and quantitative. DNA band disappears when the NP/DNA ratio is 1:16, indicating complete formation of the complexes and that the NPs have good ability to bind negative DNA.

### Investigation of binding mechanism by atomic force microscopy

**Figure 2:**
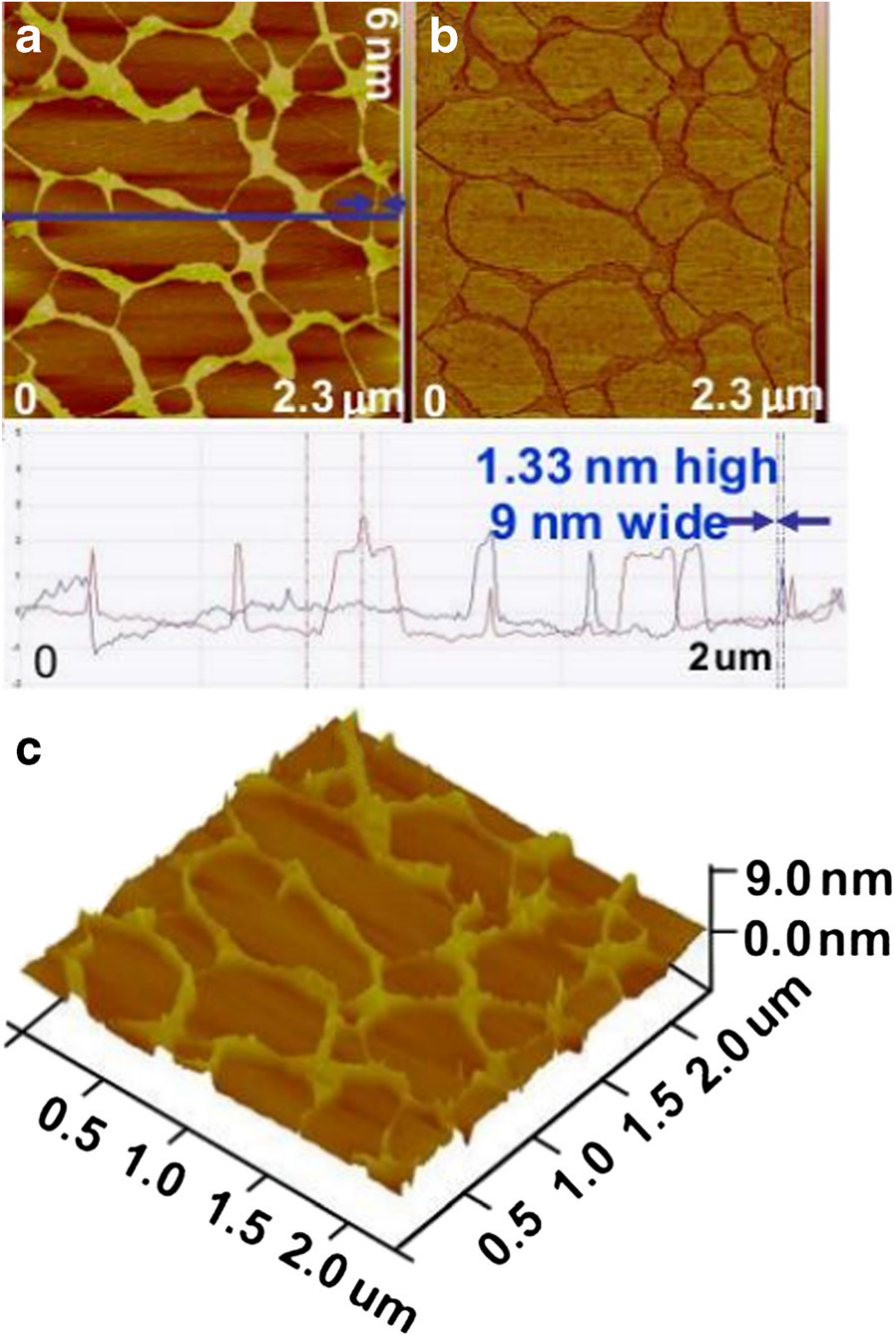
**AFM images of plasmid DNA. **(**a**) Height image (below is the corresponding topographic height profile along the blue line), (**b**) corresponding phase image, and (**c**) 3D rendering of AFM images of plasmid DNA in (**a**).

AFM experiments were carried out to investigate the morphology and microstructure of DNA, NPs, and NP-DNA complex, which is important to understand the binding mechanisms. A typical representative AFM image of DNA with relevant data analysis is shown in Figure 2a, and the corresponding phase image and the three-dimensional (3D) AFM image are shown in Figure 2b,c, respectively.

The DNA sample appears as individual DNA strands coming off of larger pieces of agglomerations with a netlike structure, which is due to the individual DNA strands which formed contacts that remain joined and form loops. As shown in the corresponding topographic height profile along the blue line drawn in Figure 2a, the results illustrate that individual thin strand of DNA is 1.33 nm high and 9 nm wide.

**Figure 3:**
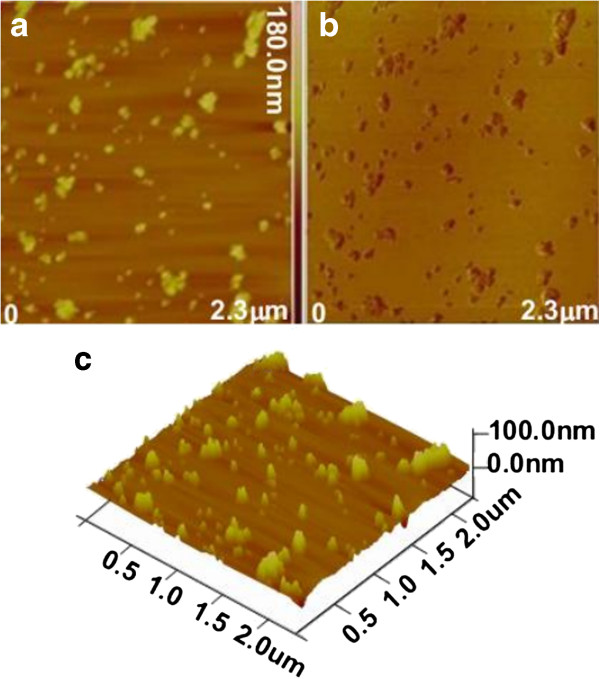
**AFM images of magnetic nanoparticles. **(**a**) Height image, (**b**) corresponding phase image, and (**c**) 3D rendering of AFM images of magnetic nanoparticles in (**a**).

AFM images in Figure 3 indicate three-dimensional topographies of magnetic fluorescent nanoparticles. It seems that the NPs have some aggregations, which may be due to the polymer matrix on the surface of NPs with too high concentration resulting in NPs becoming sticky and gluey. The particle average size of magnetic nanoparticles is about 100 nm in diameter.

**Figure 4:**
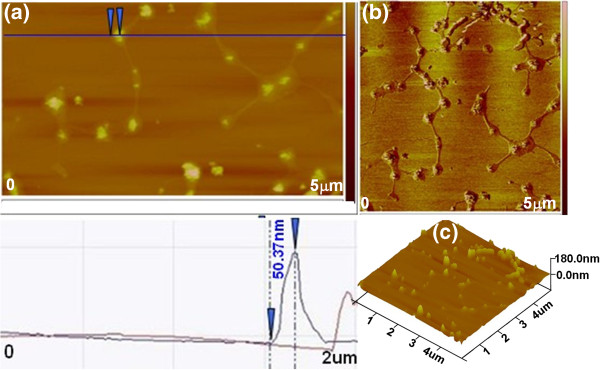
**AFM images of NP-DNA complex. **(**a**) Height image (below is the corresponding topographic height profile along the blue line), (**b**) phase image, and (**c**) 3D rendering of AFM images of NP-DNA complex in (**b**).

AFM image of the NP-DNA complex is also analyzed in order to investigate the binding mechanism between NPs and DNA. As shown in Figure 4a,b, it is apparent that several globes are attached to each individual DNA strand and interact with each other. The blue line trace in Figure 4a shows that the radius of the representative globe is about 50.37 nm, which correlates well with the size of spherical NPs. The results indicate formation of the NP-DNA complexes, which is in agreement with the agarose gel electrophoresis conclusion. The AFM images further proved an attractive interaction between NPs and DNA leading to the formation of NP-DNA complexes. As shown in Figure 4c, the 3D image of Figure 4b indicates that the NP-DNA complex surface is not smooth due to the magnetic nanoparticles attached on the DNA strand surface.

### The location of NPs in the cells

**Figure 5:**
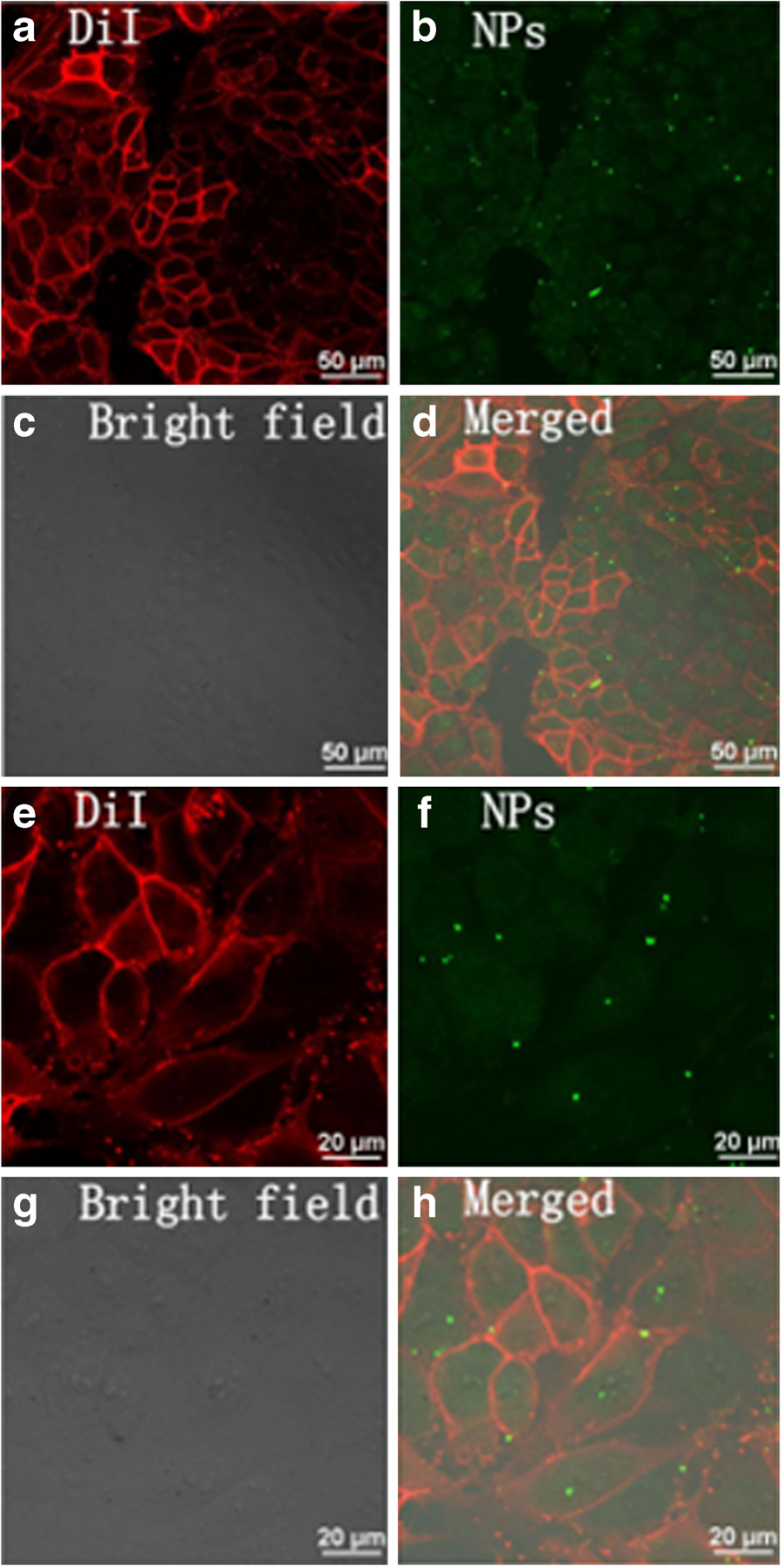
**Fluorescence images of green magnetic nanoparticles in DiI-labelled PK-15 cells and images with greater magnification. **(**a **to **d**) Fluorescence images of green magnetic nanoparticles in PK-15 cells labelled with membrane-specific red fluorescent dye DiI. (**e **to **h**) Fluorescence images with greater magnification.

To verify that the NPs can pass the cell membranes, PK-15 cells were treated with membrane-specific red fluorescent dye DiI for 10 min, and then NPs were incubated in the fluorescently labelled cells with magnetic force-induced sedimentation. After treatments, cells were dyed by DiI to show the red cell membrane location. The green fluorescence signal of NPs can be detected inside the cell after an incubation time of 30 min (Figure 5).

As shown in Figure 5a,b,c,d, NPs are internalized as intracellular green fluorescent clusters and the cell was clearly outlined with green cluster enrichment in the interior. From the images shown in Figure 5e,f,g,h with greater magnification, the location of NPs inside the cell can be observed clearer. In the process of our experiments, we found that NPs binding to cell membranes occur within few minutes under magnetic field. The presence of intracellular green fluorescent clusters was evidenced by treating NPs for 30 min, which colocalize with the membrane-specific probe DiI.

**Figure 6:**
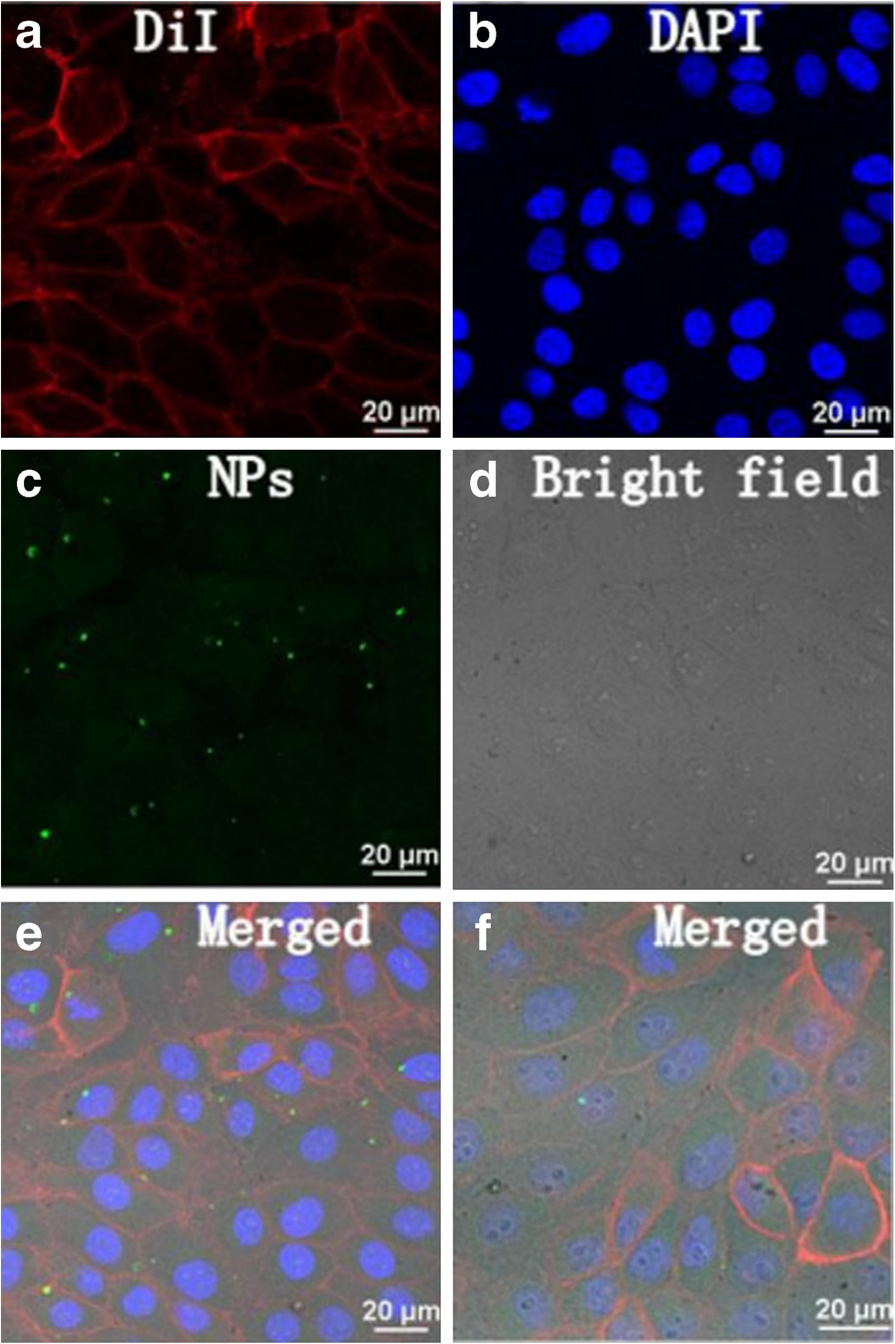
**Fluorescence images of green magnetic nanoparticles in DiI- and DAPI-labelled PK-15 cells and enlarged images. **(**a **to **e**) Fluorescence images of green magnetic nanoparticles in PK-15 cells labelled with membrane-specific red fluorescent dye DiI and nucleus-specific blue fluorescent dye DAPI. (**f**) Enlarged merged fluorescence image in order to observe the location of NPs clearer.

In order to further study the distribution of NPs in the cytoplasm and nuclei, the PK-15 cells were further treated with membrane-specific red fluorescent dye DiI and nucleus-specific blue fluorescent dye DAPI. The location of NPs between the red DiI-labelled membrane and the blue DAPI-labelled nucleus could be easily visualized in the cell. The entry of the NPs from the cell culture fluid into the interior of the cell could be readily detected. Confocal laser scanning microscopy images show uptake and distribution of NPs in PK-15 cells (Figure 6).

One can confirm both cytoplasmic and nuclear distributions of NPs in the cells, and the relative distribution in the cytoplasm was denser than that in the nuclei. From the enlarged merged image (Figure 6f), one can find that there is an overlap between the green fluorescent NPs and blue nuclei in the cell and the overlap region shows cyanic colors. It implies that green fluorescent NPs can enter the nuclei successfully as gene carrier.

## Conclusions

Green fluorescent magnetic Fe_3_O_4_ nanoparticles exhibit excellent performance as gene carrier. Magnetic nanoparticles have good binding ability with plasmid DNA. When the mass ratio of NPs to DNA reached 1:16 or above, DNA molecules can be combined completely with NPs. The morphology of the NP-DNA complex is characterized by atomic force microscopy to investigate the binding mechanism between NPs and plasmid DNA. One can find that individual DNA strand formed netlike larger agglomerations and NPs are attached to each individual DNA strand. Both cytoplasmic and nuclear distributions of NPs in the cells were observed evidently by investigating the location of NPs between the red DiI-labelled cell membrane and the blue DAPI-labelled nucleus. The relative distribution in the cytoplasm was denser than that in the nuclei. Experimental results show that the magnetic nanoparticles can pass into the cells due to good penetration ability with small size, which makes it to have the potential to become one of the more attractive gene carriers. These properties make the potential applications of NPs in animal genetics and breeding possible.

## Competing interests

The authors declare that they have no competing interests.

## Authors’ contributions

YW carried out the experimental and drafted the manuscript. YW and HC participated in the design of the study and performed the results analysis. CS, WD, JC, and XZ participated in the experimental measurements. WD participated in the cell culture experiment. HC supervised the research work and finalized the manuscript. All authors read and approved the final manuscript.

## Authors’ information

YW is an assistant professor, HC is a professor, CS is a research intern, and WD, JC, and XZ are graduate students in the Institute of Environment and Sustainable Development in Agriculture, Chinese Academy of Agricultural Sciences.
